# How Do You Touch an Impossible Thing?

**DOI:** 10.3389/fresc.2022.934698

**Published:** 2022-07-05

**Authors:** David A. Nicholls

**Affiliations:** School of Clinical Sciences, Faculty of Health and Environmental Sciences, Auckland University of Technology, Auckland, New Zealand

**Keywords:** touch, Deleuze, machine ontology, therapy, labor, work, action

## Abstract

How, and how much, physiotherapists should touch in practice is once again being debated by the profession. COVID-19 and people's enforced social isolation, combined with the growth of virtual technologies, and the profession's own turn away from so-called “passive” therapies, has placed therapeutic touch once again in an uncertain position. The situation is more ambiguous and uncertain because, despite its historical importance to the profession, physiotherapists have never articulated a comprehensive philosophy of touch, taking-for-granted its seeming obviousness as either a bio-physical or inter-subjective phenomenon. But both of these approaches are limited, with one failing to account for the existential and socio-cultural significance of touch, and the other rejecting the reality of the physical body altogether. And both are narrowly humanistic. Since touch occurs between all entities throughout the cosmos, and human touch makes up only an infinitesimally small part of this, physiotherapy's approach to touch seems paradoxically to be at the same time both highly reductive *and* ontologically vague. Given physiotherapists' much vaunted claim to be experts in therapeutic touch, it would seem timely to theorize how touch operates and when touch becomes therapeutic. In this paper I draw on Gilles Deleuze's machine ontology as a new way to think about touch. Critiquing existing approaches, I argue that machine ontology provides a more robust and inclusive philosophy of touch, pointing to some radical new possibilities for the physical therapies.

## Introduction

*How do you touch an impossible thing?*
*an imagined place, a crease in the continuity of distance?*
*How do you connect with something deemed a thing for eons, even as eons are alive in its very material?*
*How do you feel its rhythm, its time signature – a different baseline?*
*Let your imagination change what you know* (1).

If a leaf falls from a tree in autumn and, by decaying, feeds the soil, can we say that this is an act of therapeutic touch? If not, why not? Because therapeutic touch only applies to humans, we might say. But is this true? Surely, it is therapeutic when our pet dog senses some unhappiness in us and rests its head on our knee? Ah yes, but does the dog *intend* to act therapeutically? Surely, for touch to be therapeutic, it must be intentional? However, is this also true? Don't many acts of therapy happen when we touch someone without us knowing or intending their effects? So if we cannot think of therapeutic touch as exclusively human or intentional, is there *anything*—beyond the obvious differences in kind—that differentiates a massage technique like petrissage from a leaf falling from a tree? In other words, are they different ontologically? And why does this matter?

The answer to the second question is perhaps easier to resolve. On the one hand, it matters because physiotherapy practice is clearly based on some fundamental beliefs about touch^1^: it must be, because physiotherapy has historically been quite particular about the kinds of approaches and modalities that it is prepared to embrace, and those it rejects. So, the profession must have an ontological position on touch and, whatever it may be, it clearly shapes everyday practice. So, if, as one might suspect, physiotherapists rejected the idea that a leaf falling on the soil is just as therapeutic as massage to a patient's back, then we should understand why. And if we decide to think of therapeutic touch as only belonging to the human world—as most physiotherapists seem to—then we also need both a vocabulary to explain what's going on between all of the other entities that make up the cosmos, and a good reason for not including them.

At present, physiotherapy's understanding of touch is inadequate, not least because it creates some quite stark anomalies. Take, for example, the belief that manually re-inflating a collapsed lung segment can increase a patient's oxygenation. Can this be considered therapeutic touch? It is performed by a human, intentionally manually manipulating the lungs for therapeutic benefit. And yet, there is no skin-to-skin contact. The therapeutic act is performed by the air molecules forcing open and working around collapsed lung tissues. So, can we also say that air molecules are being therapeutic in their touch when they flow under an airplane's wings and keep it airborne in flight? If so, should we also consider this within the scope of physiotherapy?

In this article, I try to tackle these questions by first examining the two main philosophies that have shaped physiotherapists' view of touch in the past, before addressing their major shortcomings. I then explore Gilles Deleuze's machine ontology, and consider what this might offer to an expanded view of touch; one that may have important implications for current and future physiotherapy practice.

Before beginning, as a philosophical exploration of touch, I should say that this work is somewhat at odds with a great deal of physiotherapy literature. It has been necessary to engage in quite technical and, at times, dense language to do justice to this preliminary investigation into Deleuze's idea of touch. I have had to assume that readers will come with some grounding in philosophical concepts, like ontology, to progress the ideas here beyond the everyday and obvious. I have also assumed that readers will ecognize that the immediate application of these ideas to daily physiotherapy practice is still some way off. Having said that, philosophy is always about touch, so I hope the tone of the text is not too dislocating.

## Physiotherapy Touch in Theory

“’*To affect', ’to move'—these are simply different expressions for one and the same operation: ’to touch*”' (2).

Physiotherapy's approach to touch has historically straddled two distinct philosophies. The first is the belief that touch is a bio-physical phenomenon, conforming to Western scientific beliefs about the biology of tissue injury repair, the chemistry of atoms and fluid dynamics, and physical laws of electromagnetism and friction. This belief has long shaped physiotherapy education, with its strong focus on tissue anatomy, physiology, pathology, kinesiology, and biomechanics. It is the basis of many different forms of objective assessment, testing, and differential diagnosis, and underpins the profession's physical approach to treatment. It complements medicine's biochemical approach perfectly, and explains, in part, why physiotherapists have acquired autonomy, first contact provider status, and differential diagnostic capability in many countries.

But this approach also has some significant limitations, particularly when it comes to touch. It cannot explain the subjective experience or the meaning people ascribe to being caressed, stroked, squeezed, and stretched. During the COVID-19 outbreak, for instance, a great deal of existential dread was caused by people's inability to touch their family and friends; to reach out to others; to make contact; to be fully tactile; to be tact-full. The objective, reductive sciences do a poor job of explaining both the importance of touch as an existential human experience, but also the ways that the language and metaphors of touch pervade our cultures.

In response, a second philosophy—as old as medicine—has become increasingly important in physiotherapy. This is the belief that touch is an entirely subjective phenomenon that can only be understood by the person experiencing it. What touch means to a person, and what it ultimately achieves therapeutically, has little, if anything, to do with atoms, tissues, or coefficients of friction, and much more to do with a person's beliefs and values, personal and cultural history, uniquely lived story, and social context. “The tissues are no longer the issues”, or so its proponents say, who are now increasingly looking for ways to understand touch as a phenomenological, relational, and social experience. This approach supports the shift away from the treatment of short-term illnesses and acute tissue derangements toward the chronic, long-term, and “lifestyle” disorders that now occupy so much attention in the healthcare systems of high-income countries. Many physiotherapists now recognize that clients can often present with almost identical objective markers of disease or injury, but manifest with wildly different illness.

But this approach also comes with some significant limitations. It denies the physical reality of touch, and its concern with the slipperiness of language makes it hard to test and verify. And although biomedical approaches are also somewhat guilty of this, existential and phenomenological approaches vastly overstate the importance of the human experience, at the expense of everything else at play in the cosmos. So while both approaches can be said to be broadly humanistic, the subjectivist position is the most aggressive promoter of human exceptionalism.

## The Problem of Human Exceptionalism

“*Apes too have organs that can grasp, but they do not have hands. The hand is infinitely different from all grasping organs—paws, claws, or fangs—different by an abyss of essence. Only a being that can speak, that is, think, can have hands*” (3).

The understanding of therapeutic touch as an exclusively human phenomenon was perhaps not a conscious choice of the profession's founders when they set out to shape physiotherapy's scope and identity, but it has certainly become axiomatic to physiotherapy ever since. With the exception of the few therapists who work with animals, physiotherapy is almost entirely humanistic.

And yet, at the same time, physiotherapy has always been a practice with a strong basis in non-human entities and inorganic matter. From the biomechanical properties of gait, the therapeutic effects of heat and cold, water and sunlight, the bio-modulating effects of electromagnetic energy, and the structural effects of treadmills, stairs, and slippery floors on movement, physiotherapy is awash with different kinds of mind-independent matter. Unfortunately, physiotherapy's innate humanism tends to sublimate this matter in favor of body systems and illness experiences.

A few years ago, thinking about this problem in the context of my earlier work as a respiratory physiotherapist, I wrote an article on new materialism, and in it asked;

“*How can I reasonably practice as a respiratory physiotherapist and not have a view on the interplay between the ecology of air, the biology of breathing, the lived experience of gas exchange, the spirituality of breathlessness, or the symbiotic relationship between objects that are neither defined by what they are, nor by what they do? How can I not be interested in designer face-masks, and the creative conversion of oxygen, air and breath in works of art, or be concerned for cities like Delhi, where levels of carbon monoxide were 25 times the WHO recommended level at times last year? How can I privilege an anthropocentric view of breathing and ignore breathing as a form of anarchy, air as ’landscape', a negative space, and terra infirma? Air as terror and medium of social control? Combat breathing or muscular armor?*” (4).

Unbeknown to me, this article was published just a few months before the COVID-19 pandemic brought questions of the materiality of breathing, ventilators, oxygen supply, vapor-borne contagion, and the government of shared air, to the fore.

New materialists and object oriented ontologists argue that these intersecting, complex questions—in which touch plays such a crucial role—cannot be answered by what Graham Harman has called our tendency to undermine or overmine (5). Undermining occurs when we attempt to understand entities by reducing them down to their most elemental, fundamental structure. In the case of the body, for instance, we try to locate the atomic and subatomic particles responsible for tissue structure, derangement, and repair. Undermining is the biophysical approach that has historically dominated physiotherapy thinking about the body and touch. Alternatively, we may think of entities and matter as the product or achievement of grander social forces: as the product of language systems or discourse; as asymmetrical social power relations like class, race, and gender; or as the workings of some transcendental power like God or nature. This is overmining, and this has been the basis of the subjectivist, critical, postmodern, and existential phenomenological approaches to touch that have become more prominent in recent years. But Harman and others argue that undermining and overmining both rob entities of their unique ontological character.

Critics argue that we undermine and overmine things in order to fit them into an Enlightenment narrative that sees humans as exceptional, autonomous, and sovereign entities. We have come to believe that our conscious self-awareness and sentience sets us apart from animals, plants, and inorganic “things” in the cosmic hierarchy, and that these gifts should be the basis upon which we explain how everything works. Touch and movement, for example, are judged against a human scale and not, for instance, on the kinds of geological time scales that see mountain ranges form and move to shape the landscape we all live upon. Our light, agile bodies become the normative measure of touch and movement, against which the slow creep of mountains appears incremental and dull.

The problem of how to think about touch in a more inclusive way, then, becomes a vexed question. How should we proceed, because the biosciences certainly offers us some of the tools to appreciate the mind-independent reality of objects and a range of methods to empirically investigate them but, at the same time, they lack an understanding of the lived experience of things? Equally, subjectivism, critical theory, postmodernism, existentialism, and phenomenology gives us access to the existential experiences of things, but currently offer little beyond human understanding?

Some have argued that the best approach might be to take the best of the scientific *and* experiential philosophies, and think about touch more holistically. And we have seen some of this in both the emergence of pragmatic bio-psycho-social, integrative medicine, and motor intentionality approaches in physiotherapy in recent years (6–9). This hybridization or slurring of philosophies suggests that physiotherapists have become dissatisfied with the way touch has come to be understood, both within and without the profession. But both of these approaches have their problems.

The first is that more pragmatically “holistic” approaches can only change practice at a superficial, experiential level. And while they allow some physiotherapists to claim all manner of adapted, hybrid roles and responsibilities, they also create all manner of confusion about the underlying ontological framework now at work in the profession. Where once, for instance, physiotherapists were consistent in their belief that pain, illness, and injury resided within the body, now it is not so clear. And advocates of new bio-psycho-socially orientated programs have been somewhat coy about identifying where things like pain now actually reside. Of course, this has enormous implications for physiotherapy education and practice, because if we no longer need to look for pain, illness, and injury in the body, we can dispense with the anatomy, physiology, and pathology that has always been at the heart of professional training. But again, advocates have been somewhat shy in suggesting this, perhaps because they know that this will inevitably lead to us redefining where we believe health actually resides, which in turn will result in us reshaping the profession's relationships with others, including the medical profession and the broader “state” and, in so doing, undermine our long-fought-for social power and prestige. Perhaps not surprisingly, advocates of new, more inclusive approaches to physiotherapy, have been reticent about going this far, suggesting the full ontological implications of their ideas have yet to be worked through.

Similarly, those who have argued that physiotherapy should turn away from so called “passive”, touch-based therapies have done little to indicate whether physiotherapy should fundamentally change, or we should simply be changing our professional wardrobe to fit the current fashion. If the argument is that there is no evidence for the efficacy of touch-based therapies, we once again return to the question of how touch is defined in the first place. And, as already stated, this assumes a great deal about the nature of touch in physiotherapy that has never been thoroughly articulated or examined. So, given all of the limitations of existing approaches to touch, and our lack of engagement with the subject at an ontological level, the following sections explore the machine ontology of Gilles Deleuze, which has touch at its core.

## Deleuze's Machine Ontology

“*[E]ven with habitable planets orbiting one tenth of all stars, the fraction of living matter in the universe is about one-billionth of one-billionth: If all the matter in the universe were the Gobi Desert, life would be but a single grain of sand*” (10).

Although this is often said when speaking about great minds, Gilles Deleuze (1925-1995) is considered by many as one of the foremost philosophers of the last century. His eclectic, iconoclastic, often opaque writings, produced from the 1950s until his death, (by suicide, perhaps as a result of worsening lifelong respiratory illness), have set him apart as both a polarizing figure in French continental philosophy, and as a genius of unparalleled inspiration. Deleuze's four main books— Difference *and repetition; The logic of sense; Anti-Oedipus*; and *A thousand plateaus* (11–14), sketch out a way of understanding entities, objects, matter, and things in the universe—whether real or imagined, alive or dead, past, present or future—that some argue resolves many of the issues created by scientific rationalism and humanism. The approach is called machine ontology, and it holds a central place for our understanding of touch.

Before attempting to sketch out the main features of machine ontology, it is worth noting that because it offers a radically different view of the nature of reality, it will be difficult to summarize here in language that will be conventional or comfortable for most readers. Deleuze notoriously used conventional terms like “machine”, “the body”, “sense”, “desire”, and “relations” in unconventional ways, and much of his writing was an attempt to not only think against convention but to also find new modes of expression. The key principles of machine ontology are clear, however, so readers looking to understand more of the details of Deleuze's approach will find excellent summaries in a number of secondary texts as well as in Deleuze's own writing (15–19).

Firstly, Deleuze asserts that all entities are machines. Not metaphors of machines, but actual machines. These machines do not exist “for us”. Nor are they the reflections or expressions of anything else: be it God, spirit, science, substance, life-giving vital impulses, genomes, consciousness, social power relations, discourses, laws of nature, cognition, neurons, or subatomic particles. Machines require no support from anything else, and can neither be reduced to their parts (undermining), nor to their context (overmining).

Deleuze then tells us that entities can be anything: knife rests, hotel chains, rivers, circuses, hydrogen atoms, Beethoven symphonies, rocks, ideas, cities, computer games, packs of rats, atomic bombs, physiotherapy, tornadoes, Harry Potter, a game of tennis, fear, a riot, thought, William Shakespeare, people, Netflix, … and all of these share an equal claim to reality. Alive or dead, historical or in an imagined future, mind-independent or sub-conscious, material object or concept. They are all ontologically equal.

To define the differences between entities and explain how they come into being, remain as they are, communicate, shift, and change, Deleuze constructs a fourfold structure shared in common by all things. This is what Deleuze means by *repetition*—that all things share the same ontological structure. On the one side of this fourfold are real properties, possessed by every entity, that are always withdrawn and inaccessible. These are what make a cloud a cloud despite its multitude of shapes, molecular structures, colors, and locations in space. Real, mind-independent properties make up what Deleuze calls the virtual real, and Deleuze believes these properties exist even when all of the entities relations and surface appearances are stripped away. They always exceed the ability of another entity to know them. So, no matter how scientific our empirical investigations of an entity are, we will never exhaust what the entity can be or do.

At the heart of every entity is the first component of Deleuze's fourfold, what he refers to as an entity's body. This is not the kind of body we refer to in physiotherapy and Western healthcare, but a philosophical notion of a bare, non-relational unity that exists outside of all relations. The body of an entity never integrates with the entity's qualities and remains aloof. In this way, the surface qualities of an entity (it's color, size, texture, taste, etc.) can change, but the apple remains an apple; a human being remains a human, despite differences in body composition, shape and size; and a dream of flying remains different from a film showing someone dreaming about flying. Deleuze calls this irreducible body the *Body without Organs*. As mentioned just, this is not simply the corporeal body of a person containing various bodily organs, but a body at the heart of every machine; a body that is unique to that entity, possessed by each and every thing, and a structure that never accounts for the diversity of qualities or relations the entity engages with. It is a “withdrawn” body: a body “without” any “organs”.

The second component of an entity on the virtual side of the fourfold is its desire. This is not the kind of desire we normally associate with wanting something we lack, but rather a machine's stationary, unconscious motor; its factory, its “being”, and the ground upon which all its relations and manifestations are built. Desire still resides in the virtual real half of each entity's fourfold ontological structure, and is necessary to complement the Body without Organs, which is empty. When two entities touch, a new “third” machine is always automatically created and, in being created, it immediately possesses a body (to define this entity as this), and desire (to differentiate this entity from that). As Deleuze says, if the body is the egg, desire is what fills it. Desire defines what an entity has, and so provides the ground for what the entity can do.

In explaining desire, Deleuze talks about an entity possessing an Idea (always capitalized), singularities, and a code, which “fill” the virtual real and give the entity its distinctiveness. For instance, a person's skin “wants” to have a certain texture, color, and stretchiness in order for it to be skin and not, say, a rock, a breath of wind, or a mood. It's desire is to be filled with properties that allow it to be achieve its skin-fulness. These qualities might be different for different people, but the desire to be skin remains the same. The same is true for all entities whether they be therapy assistants, ACL ruptures, or calcium channels. For any entity to possess surface properties and relate to other things, it must possess a deeper desire that is the motor for the entity being what it is, and not something else.

Crucially, though, Deleuze argues that the virtual side of each entity never fully manifests in relations and always remains a private reality. What we access when we encounter another entity—what everything in the cosmos encounters—is a machine's qualities and through that its “sense”.

An entity's actual, relational properties, through which it comes into contact with others, resides on the other side of the fourfold to the Body without Organs and desire. How an entity is created, shaped, and destroyed, only happens through relations. But relations alone cannot explain the nature and diversity of being in the cosmos (a key criticism of new materialism), because if everything necessary for change had to be present in relation, there would be no surplus from which mutation, creativity, deformity, and spontaneity could emerge. Hence, why entities must have a private interior that always exceeds what is available to connect with others.

But when we encounter another, we sense its unity: we see a tree, or a cloud, or John Lennon, and know it to be this and not something else. This unity derives not from the entity's qualities, but from what Deleuze calls “sense”—the third structure in the fourfold. A tea cup might look differently if I close the curtains and block out the light (a change in the entity's qualities), but it remains a tea cup. Similarly, a patient with a stroke might walk differently in on a clinic floor than they do at home, but they remain the same person. So as well as manifesting different qualities, every entity also possesses a relational “ground” or “sense” upon which the entity's qualities are arranged. If all of an entity's qualities were stripped away, sense would be the framework left behind that defined how the entity might be comprehended. As with the Body without Organs and desire, it is produced immediately as a new entity comes into being. It is the ability of an entity to comprehend another and be comprehended. It is immanent to relations but not the relations themselves, which can be pushed and pulled depending on the entities involved. Like the Body without Organs, it is neutral, sterile, aloof, and indifferent. But it is always there.

The fourth and final component of Deleuze's fourfold are an entity's qualities: those surface features that distinguish a treatment table from a kitchen table; a rock from a rock song. Qualities are always in a constant state of flux and flow, but still remain anchored to the entity's sense. In this way, an entity becomes recognizable and trees don't suddenly start sprouting lampshades and Greek tragedies. The constant ebb and flow of an entity's qualities is continually being broken in upon (touched) by other machines, and when this act of intrusion is successful, a new machine is created. This new machine always has a shot at altering the desire of the machine it invades, and thus changing the nature of the world as it goes.

This process of change, mediation, connection, and destruction is defined by Deleuze by three distinct “passive syntheses”. Connective syntheses is the first, and describes how entities relate to one another. The second, or disjunctive synthesis, describes entities “register” one another. And the third, or conjunctive synthesis, defines how new entities are formed when they touch. Deleuze calls these “passive” syntheses because there is no driving force or guiding hand dictating what or how entities change (Remember, Deleuze does not believe any entity can be reduced “down” to smaller elemental particles, or “up” to a higher authority). But more than this, Deleuze argues that the *parts* of a machine are irreducible to the machine itself and to each other, and that even an entity's surface relations and manifestations are irreducible to its body and its desire, which always withdraws and exceeds what an entity appears to be. This is what Deleuze calls the radical difference of all things—the difference between an entity's real, virtual, private interior, and its actual manifestations in relation—a difference shared by all things (hence the title of his book *Difference and Repetition*).

Entities in the universe can only come to know other entities through contact with their manifestations and relations. Because entities cannot be reduced to God, consciousness, nature, or any other “higher” being, everything that happens must happen between entities themselves. This is Deleuze's radical pluralism. Entities never collapse into each other or “merge”, and only ever generate a swarm of new beings (explaining why there is so much of everything in the universe). They can, however, come together into contiguous assemblages or rhizomes, forming social and technical machines in which entities “come under the spell” of the molar assemblage (in symbiotic relationships or nation states, for instance).

Unlike many of his postmodern, new materialist, and speculative realist peers, then, Deleuze does not advocate for a flat ontology between all things. His machine ontology is strongly hierarchical. But it is not based on beliefs in God, nature, science, or other transcendental forces. True to form, though, Deleuze does try to confuse matter further by naming his methodology for interrogating entities *transcendent empiricism*. It is empiricist because he believes that we can only understand what entities are able to do by studying the signs they create through their manifestations and actions. And it is transcendent because he believes that thought can provide clues to the radically withdrawn formal nature of entities that can never be wholly grasped.

So none of us, helium balloons or humans, can ever fully know what another entity is, or what it can do. Hence, Deleuze dispenses with our desire to know what things are, and focuses instead on what things can do (The question “what is touch” would be very un-Deleuzian in this respect). Deleuze does believe it is possible, however, to progressively deduce what an entity can do from its relations and signs. Thus acts of touching, seeing, colliding, pulling, having, knowing, crushing, rubbing, placing, destroying, pointing, recruiting, forcing, and treating, are all tools we can use to tell us something about the entities involved.

## Machine Ontology in Context

In many ways, Deleuze's machine ontology echoes many of the claims made by Aristotle in *De Anima*, perhaps the first extended philosophical treatise on touch. It was Aristotle that called touch the “mother” of all senses, recognizing that it was the first to develop *in utero*, the only one of the five senses that involved the whole body, that it was the only sense in which humans surpassed animals, and that the other senses were all specializations of some form of touch concentrated around the head; “All living beings possess touch and every sense implies tactility of some kind: light strikes the iris, sound the tympanum, odor the nose buds, taste the tongue” [(20), p. 35, see also (2, 21)]. Touch is also the only one of the common animal senses that is reciprocal: one can never touch and not be touched back.

Other approaches to understanding touch: as sinful or as a bio-physical phenomenon, emerged over the next two millennia, but *philosophical* thinking on touch only really re-emerged in the late 20th century. Mirt Komel suggested recently that until 2005, and the publication of the English translation of Jacques Derrida's book on the writings of Jean-Luc Nancy, which claimed Nancy as the first modern writer on touch (22), and Constance Classen's edited compendium (23), there had been no concentrated attempts by philosophers to deal exclusively with touch [(24), p. 1].

Touch has, of course, been an important *component* of philosophical thinking, particularly in phenomenology, where Heidegger, Husserl, and especially Merleau-Ponty, saw touch as a vital part of how we come to “being”. And phenomenology has perhaps been the most active philosophical school contemplating touch ever since (25–27). For example, Deane Juhan, drawing heavily on the work of Merleau-Ponty [(28), p. 231], has suggested that “[t]actile experience tells me as much about myself as it tells me about anything that I contact” [(29), p.34]—an argument Deleuze will strongly refute below. Through touch, Juhan argues that “[b]y rubbing up against the world, I define myself to myself”, and learn that “I am more cohesive than water, softer than iron, harder than cotton balls, warmer than ice, smoother than tree bark, corset but fine silk, more moist than flower” (ibid).

But in every case, touch is seen from the perspective of the human. In his 2013 book *The first sense* (30), Matthew Fulkerson argues that “our” thinking and research had for too long been oculi-centric; over-stating the value of sight in the way we research, study, and think about the world around us. Fulkerson suggested that there is much to learn about the world from our contact with it, from tactile data, “digital” discourse. But Deleuze's machine ontology takes this argument much further, suggesting that our focus on judging the world by what we see, has been a powerful driver of human exceptionalism, to the detriment of all of the other forms of touch going on in the world. And the recent upsurge of interest into the meaning and significance of touch *by “us”, for “us”* only bears this out.

But if we accept Deleuze's premise that entities cannot exist without the capacity to touch others, and that all something can ever know about another comes through its limited contact with its surface manifestations (sense and qualities), then it follows that everything that can exist in the cosmos is the direct result of touch. Everything from works of fiction, hamstring muscles, clouds, crowds, hospital buildings, and media personalities owes a debt to touch.

It also follows that since contact with the surface of another is all an entity has if it is to “have a shot” at altering another entity's desire, it makes sense that every thing in the cosmos maximizes whatever tools it has at its disposal to make contact, feel, touch, encounter, and palpate another. Recent studies from art theory to zoology have revealed tactile sense in all manner of organic entities (31–34), but just as fungi palpate the soil, the soil, in its own way, palpates back. Humans have their five highly evolved senses, and have used these well, but pencil sharpeners, swordfish, and the poetry of Emily Dickinson, must also have their own capacities to touch and be touched, even if they are different to the ones “we” know and are familiar with.

Mention of Emily Dickinson's poetry is a reminder that for us to fully grasp the myriad ways entities—which, again, includes *all* things: living or dead, thought or object, organic or inorganic—use touch, we need to remember that touch can take many forms. Think here, for example, of the numerous ways metaphors of touch shape our everyday contact with others: we are tactful; we make contact with people; we feel another's pain; we hold thoughts and strong beliefs; some people are hands-on managers; we watch moving films; we feel under pressure, and are pushed for time. This form of touch “touches the untouchable” [(22), p. 18], as both the tangible and intangible are always the object of touch. If humans have made such varied and interesting uses of some kinds of touch, what touch capacities must be present in glaciers, air molecules, songs, pollen, and viruses, for them to exert their differential influence in the world?

Thinking about touch from a human perspective may, therefore, seriously limit our ability to see what touch is actually capable of. The kinds of bio-physical approaches to touch that have formed the backbone of physiotherapeutic practices over the last century have limited philosophical utility because they have still to address the question of what touch actually *is* (And while this is not a question Deleuze is interested in, it certainly is part of the raisin d'être of the biosciences). When someone's hand touches another person's body, what is it that actually touches? At an atomic level, we know that the relative distances between atoms is so vast that there is much more empty “space” within structures than actual solid substance. So, if anything actually “touches”, from a bio-physical perspective, it is perhaps not atoms at all. Flesh never actually encounters flesh. Even the idea of boundaries between one thing and another that the biosciences rely on so implicitly are fraught philosophically, and there are many examples of how it is impossible to tell where anything begins or ends in real life [see, for example, Achille (35)].

Similarly, the claims of the experiential philosophies to explain touch fail because they only tells us about touch from a human perspective, and cannot account for gargantuan, overwhelming, and vastly more common forms of touch going on in every corner of the universe all of the time, beyond the reach of human experience. So if we make existential, linguistic, discursive, critical, or phenomenological thinking the cornerstone of our understanding of touch, we may well be left wondering how to explain everything else going on. If being beholden, tactful, making contact, surfaces, and digital, are specifically human subjective experiences, how should we understand all of the other forms of touch? And if experiential philosophers allow us to extend our use of these terms to fungi, trains, fictional characters, and swarms of wasps, how will they hold on to the subjective uniqueness of human experience?

Although these may seem somewhat abstract questions, suggesting that touch is a world so vast that it is both incomprehensible, and so vague as to be of no relevance or importance to physiotherapists, these questions are absolutely at the heart of physiotherapy practice because not only do they help us understand how we currently understand touch, but also how the scope and scale of our practice might change if our view of touch was radically changed.

## Implications for Physiotherapy

“*While unconscious creation—animals, plants, crystals—functions satisfactorily as far as we know, things are constantly going wrong with man*” (36).

Despite the fact that “there is no shortage of theoretically-driven or empirically-informed accounts of touch” in areas like anthropology, archaeology, architecture, art history^2^, botany, computer science, cybernetics, education, experimental psychology, haptic media studies, haptic sociality, linguistics, literary theory, neuroscience, and sociology (24, 37–41), there is a good case to be made that if any group in society should have a thoroughgoing philosophy of touch, it ought to be those who deal in the physical therapies. And certainly some have tried. Deane Juhan's eloquent handbook for bodywork being perhaps the best example (29)^3^. But physiotherapy has always historically taken a narrower view, confining itself to bio-physical understandings of touch or, more recently, Nordic phenomenologies inspired, most especially, by the works of Merleau-Ponty and van Manen (6, 43–45). Of course, other theoretical aspects of touch are periodically discussed in the physiotherapy literature (46–53), but the emphasis always remains firmly grounded in the humanistic.

And yet, as I have argued, the human experience is only one very small facet of universal touch, and is, indeed, only one small facet of the myriad forms of touch that make up the physical therapies. By relegating everything that does not directly involve human experience to a supporting cast of objects, matter, and things, whose only value is in the ways it can function “for us”, physiotherapists have limited themselves to a very restricted philosophical palette with which to understand therapeutic touch in its entirety, including the myriad ways that mind-independent therapeutic touch operates.

As professionals with a special interest in the workings of touch, the sheer emancipatory potential of Deleuze's machine ontology is staggering. No longer does touch need to be limited only to person-person interactions, but can embrace the full panoply of entity-entity contact and connection. Machine ontology opens up the possibility for entirely re-interpreting the scale and scope of physiotherapy practice.

As I mentioned earlier, I have been attempting for some time now to think about my interest in respiratory physiotherapy as an assemblage of all manner of entities that I previously hadn't considered, deliberately avoiding the traditional privilege given to the patho-anatomy of lung disease, the technical management of acute and chronic respiratory failure, or the subjective experience of breathlessness.

But this is not merely designed to help me think beyond the silos of biomedicine, existentialism, or structuralism, though. Because some readers might argue that a more inclusive “holistic” view of oxygen, air, and breath, could be achieved by adopting a bio-psych-social (BPS) approach to practice. However, the BPS approach and model—along with all supposedly “holistic” models—suffer because they achieve the false impression of harmony by ignoring the fundamental ontological differences between their components^4^. What results is a practically pleasing appearance of inclusivity. But the cost of its pragmatism is a vague and over-simplified understanding of everything.

Deleuze's machine ontology takes the exact opposite approach. Rather than arguing that there is one over-arching, transcendental whole, into which the bio, psycho, and social feed, Deleuze proposes a radical pluralism that allows us to study the anatomy of the alveoli and the physiology of gas exchange, as well as the social cost of urban pollution, the climate effects of deforestation, and the pleural assemblages people form with ventilators and air conditioning units, without squeezing one ontological reality into another, or losing the radical difference that exists between all things.

Returning to touch, though, there are many ways that machine ontology could be used to look differently at the kinds of touch at work in the world and, in doing so, radically reframing our thinking. For instance, exploring the deprivation people have reported during COVID-19. Concerns have emerged over people's stress, their physical isolation, people's levels of pain and discomfort, the loss of social bonds and the breakdown of traditional customs and rituals, loss of attachment and our ability to maintain intimate relationships (54). At the forefront of these debates have been psychologists concerned with the effects of the loss of close physical contact on people's mental wellbeing, and phenomenologists concerned with injury to the sovereign role played by relationships in people's sense of being. But in privileging direct human-to-human contact, what neither of these approaches account for is the fact that people were in touch with the world just as much during the COVID-19 outbreak, with all its restrictions on human intimacy, as they were before or after. Since there is no existence without touch—the touch of clothes on the skin, the touch of oxygen molecules in the hemoglobin, the touch of light from the computer screen onto our retinas—to make so much of the physical dimensions of human skin-to-skin contact at the expense of *everything* else sounds more like an appeal to humanism than an accurate appreciation for how touch is operating in and through COVID-19.

So, COVID-19 certainly revealed the latent humanism at work in a lot of our thinking about touch. But it also showed was how much health services had become overly dependent on physical contact to function, and how necessary it was for people to have access to a wide network of other forms of help and support if they were to survive and thrive. What became evident was that, like many other traditional health services, the provision of hands-on (physio) therapeutic touch had been undergoing a decades-long de-professionalization that the COVID-19 outbreak merely exacerbated (55). Where bio-physical and phenomenological understandings struggle to understand these socio-political drivers of practice, machine ontology can help. By expanding our understanding of what touch is, and the role it plays as the medium of engagement between all things, there is no reason why physical therapists cannot make touch one of the new frontiers of planetary health, shifting research and practice away from an exclusive focus on human interests, and driving new thinking into the nature of what really “matters” in the world.

The thought that there may be more therapists “out there” in the world than we had previously acknowledged, or even been able to imagine, is a challenging thought. But clearly, therapeutic touch cannot be retained as an exclusively human trait when algae are frantically absorbing atmospheric CO2, Greta Thunberg's words are inspiring people to change, fungal spores are reforming soil after devastating forest fires, and cycle lanes are getting people out of their cars. This is not to romanticize nature or see touch as a purely ecological problem, but rather to suggest that there is more going on in the world than merely “the human touch”.

But such emancipation also comes with its own problems, and these need to be considered, too. How, for instance, would physiotherapists differentiate themselves from others if their particular subject of special interest was a feature of every cosmic encounter? What would *not* be part of (physio) therapeutic touch? And how would we distinguish forms of touch that are therapeutic from other, incidental, or harmful forms of contact? As the title of the article suggests, how would we touch this seemingly impossible thing?

The first question is really one of politics, not philosophy, and was approached recently in the book *Physiotherapy Otherwise*, where I argued that physiotherapists and others were now entering a post-professional era in which professionals would become de-centered from areas like healthcare and education (55). Rather than fighting against this trend and looking for ways to re-claim traditional notions of touch, I argued that physiotherapists should be working actively to de-colonize their practices and break down the traditional protective enclosures that have afforded them so much privilege and prestige in the past. Part of this centered on re-enchanting therapeutic practice, and locating the Deleuzian intensities (another word for assemblages or rhizomes) at the heart of the physical therapies.

The second problem, though, is distinctly philosophical. Returning to the question at the beginning of the article; if a leaf falls from a tree and feeds the soil, can we say this is a therapeutic form of touch? If not, why not? And how do we distinguish “good” touch from bad, harmful, or abusive touch, if touch is seen only as a universal factory of machine production? From a Deleuzian perspective, this is much harder to answer. Firstly, Deleuze would not accept the idea that the concept of “therapy” could pre-exist as an unchanging essence sitting above or outside relations, shaping its direction. For Deleuze, no such transcendental ideas exist. So therapeutic acts must happen during the acts of touching, seeing, colliding, pulling, having, knowing, crushing, rubbing, placing, destroying, pointing, recruiting, and forcing, that make up entity-entity relations. Therapeutic effects would lie within the three passive syntheses that Deleuze uses to describe how entities relate, register, and retain one another. And rather than relying on some external arbiter (God, science, morality, nature, etc.), to determine that this or that act is or isn't therapeutic, Deleuze might suggest that any act that increases the possibility for more diverse and pluralistic forms of touch should be considered a good thing.

The idea that touch might be seen as good or bad is also an ontological problem, not a moral concern for Deleuze, who would see all efforts to impose such values on to touch as a naked attempt to re-impose a transcendental humanism—the very thing his philosophy attempts to counter. Instead, Deleuze would see “good” forms of touch as those that opened spaces for deterritorialisation, movement, a thousand new machines, and a thousand new connections, and “bad” forms being those that closed off possibilities, territorialised, stifled, suffocated, or collapsed possibility.

This ontological position has come in for some criticism in recent years, because of the seeming absence of a strong, overt political position toward things like social justice and planetary health. Should we not, Thomas Lemke's argues, take a stronger position on human action, given how destructive it has been to human and non-human life on the planet? Should philosophies like new materialism and speculative realism offer a “more convincing way of addressing the issue of ontological politics” [(56), p. 78]? In Lemke's view, the rejection of human exceptionalism, and the desire to “theorize reality independently of however human beings may experience it” (15), provides nothing to help us overcome prejudice, social injustice, hatred, discrimination, and abuse, never mind climate change, the rampant effects of late capitalism, and global political instability.

Others might disagree, though. Margrit Shildrick, for instance, has written that touch is the very thing that signals potential danger in a specular normative economy that privileges separation', because it holds the potential to disrupt the male gaze that has sought “mastery over all things external to him, whilst at the same time looking into himself for knowledge of his own being” [(57), p. 388]. Here you have both the critique of our biophysical *and* inwardly humanistic and androcentric approaches to touch that have dominated physiotherapy and the health sciences to date, in which “the emphasis on the detachment… at the expense of the immediacy of touch is a characteristic of a masculinist logos” (ibid). Shildrick's argument is that touch holds the potential to collapse the distance between self and other, disrupt logics of normal and anomalous, and address the very asymmetries that have created the need for restorative therapy in the first place. Perhaps, then, an appraisal of touch through a Deleuzian lens is long overdue.

## Conclusions

Throughout much of its history, physiotherapists have unquestioningly viewed therapeutic touch as a bio-physical phenomenon, in keeping with the profession's affinity with treating the body-as-machine. Recent years have seen the emergence of interest in the phenomenology of touch, as physiotherapists have embraced more person-centered and (inter-) subjective approaches to practice. This has undoubtedly given physiotherapy a more expanded view, but therapeutic touch remains devoutly humanistic. At the same time, in philosophy, the emergence of Gilles Deleuze's machine ontology has coincided with an enormous growth of interest in objects, things, and matter. At the heart of this philosophy is the concept of touch, which should make it an ideal framework for those working in the physical therapies.

Deleuze's approach turns away from both the bio-physical and existential, humanistic approaches common to physiotherapy and, instead, offers a radically pluralistic view of entities and a fourfold structure shared by all. The structure differentiates that which is the private interior of an entity—withdrawn, neutral, and irreducible—and the manifestations and relations through which all entities connect. Deleuze's three passive syntheses then explain how entities relate, register, and retain one another.

Deleuze's machine ontology is both philosophically and politically radical for a profession that has long aligned itself with biomedicine. But times are changing, and many physiotherapists are looking for new ways to think about and construct their therapeutic practices. Many of the responses emanating from within the profession lack a strong philosophical justification, and so risk being merely short-term, pragmatic solutions to the most immediate concerns. However, rarely in physiotherapy practice, never mind life in general, are short-term pragmatic solutions desirable or effective. To think more radically—in the sense of relating to the very *roots* of the problem—may require a deeper appreciation for the philosophical bases of our practices. And little can be more fundamental to the practice of physiotherapy than therapeutic touch.

Machine ontology is not without its problems, however. It comes with no easy formula for how to apply it. Its concepts and language are idiosyncratic and often confusing. It dissolves boundaries around professions rather than solidifying them. It has no interest in defining what touch or therapy are, and assumes they are only generated in relation, so have no permanent, transcendental basis. And it appears to offer little, yet, to help us address some of the worst aspects of human exceptionalism. That being said, machine ontology may just provide a 21st century philosophy that can finally emancipate therapeutic touch from the shackles of undermining, overmining, and human exceptionalism.

## Author's Note

Touch is not only the “mother” of all senses, but it was the first to be thoroughly studied when Aristotle wrote *De Anima*. Therapeutic touch plays a vital part in the physical therapies, but these approaches tend to privilege two mutually exclusive views of what touch is. It is seen as either a biological and physical phenomenon, or a subjective human experience. Neither of these approaches are satisfactory, though. In this article, I offer the first analysis of touch using Gilles Deleuze's concept of machine ontology. Deleuze believed that touch lay at the heart of all entities, and offered a radically different way to view “things” in the world; a way that holds enormous potential for an expanded view of the physical therapies. This article explores the theory and speculates on ways it might change thinking and practice.

## Data Availability Statement

The original contributions presented in the study are included in the article/supplementary material, further inquiries can be directed to the corresponding author.

## Author Contributions

The author confirms being the sole contributor of this work and has approved it for publication.

## Funding

Funding to support open access publishing was provided by AUT's School of Clinical Sciences PBRF Open Access Publication Fund.

## Conflict of Interest

The author declares that the research was conducted in the absence of any commercial or financial relationships that could be construed as a potential conflict of interest.

## Publisher's Note

All claims expressed in this article are solely those of the authors and do not necessarily represent those of their affiliated organizations, or those of the publisher, the editors and the reviewers. Any product that may be evaluated in this article, or claim that may be made by its manufacturer, is not guaranteed or endorsed by the publisher.

## Footnotes

^1^I have referred to physiotherapy as a unified professional entity throughout the paper but recognize that this is, at best, only a convenient shorthand term for a profession that has many facets and forms of expression. Readers will, I hope, find that the way physiotherapy is made plural through Deleuze's work does justice to its diversity.

^2^Beyond the role of touch in sculpture, perhaps one of the greatest discussions about touch in art history has revolved around Michelangelo's intention in painting God almost, but not quite touching Adam to spark life into humankind in his frescoes on the Sistine Chapel ceiling c.1508-1512.

^3^In many ways, although it is deeply phenomenological, Juhan's writing on bodywork comes closest to approximating machine ontology. For example, Juhan suggests that: We are “forms which are continually undergoing formation and re-formation. We are objects, and there is every reason to suspect that the processes of matter within us are no different than natural processes anywhere.” And this: “We are, of course, mechanical in so many of our physical aspects, so there is a great deal of justification for focusing upon these sorts of effects and explanations, as far as they go. But we are much more than mechanical. We are a confluence of physics, chemistry, and consciousness, streams and quanta of energies that interpenetrate one another in enormously complex ways, that moment by moment create layers of effects, and in which the subtle and gross are always inextricably intertwined” [(29), p. xxiii]. Despite these inferences though, the phenomenological underpinnings of Juhan's work always pull him back to thinking about these many entities as only having significance when they function “for us.” In a similar way to Juhan, Alice Whieldon and Akinobu Kishi argue that “The real meaning of touch is making natural, easy, human contact” (42). But Deleuze would suggest this ignores a galaxy of other touch going on all the time; touch that is no less therapeutic than the touch being performed by us and for us, and with which humans have very little involvement.

^4^In the case of the BPS, the psych- and, to a lesser degree, the socio- elements almost always remain as subordinate conditions of the bio-.
